# Vacuum-Assisted Closure Device as a Suction Tool in First Stage Autologous Auricular Reconstruction

**DOI:** 10.1007/s12663-025-02884-8

**Published:** 2026-02-21

**Authors:** Mina Asaad Sawiris, Ibrahim Kamel, Mohamed Samir Badawy

**Affiliations:** Department of Plastic, Burn and Maxillofacial Surgery, Ain Shams University, Ramses Street, Cairo, 11591 Egypt

**Keywords:** Microtia, Patient satisfaction, VAC, Ear, Negative pressure

## Abstract

**Background:**

Autologous auricular reconstruction typically involves the fabrication and insertion of a costal cartilage framework. Achieving a natural contour requires the skin envelope and cartilage framework to remain in close touch. Traditional suction drains might not be able to sustain a constant negative pressure, leading to suboptimal outcomes. This study evaluates the novel use of a vacuum-assisted closure (VAC) device as a suction tool in the first stage autologous auricular reconstruction.

**Methodology:**

Twenty patients; with unilateral microtia underwent autologous ear reconstruction using costal cartilage graft were selected, 10 patients used a syringe as a traditional method for suction while 10 patients underwent reconstruction with the incorporation of a VAC device to provide an uniform negative pressure and promote adherence of the skin flap to the underlying structure. Outcome measures included skin flap viability, contour definition, complication rates, and patient satisfaction.

**Results:**

Patients reported high satisfaction rates with early aesthetic outcomes higher for the VAC group (22 ± 1.49) than syringe suction (18 ± 1.17) (*P* < 0.001). Also, 9 out of 10 patients (90%) in VAC group didn’t record any complication while 30% of Syringe patients had complications like hematoma, skin necrosis and exposed cartilage. Use of the VAC device resulted in improved skin adherence and enhanced definition of auricular subunits.

**Conclusion:**

The VAC device is a safe and effective tool for optimizing surgical outcomes in the first stage of autologous auricular reconstruction. Its application promotes consistent contouring, reduces complications, and enhances patient satisfaction. Further studies are warranted to validate long-term benefits.

## Introduction

 Auricular reconstruction remains one of the most challenging procedures in reconstructive surgery despite the ongoing advances in surgical procedures and suction tools [1]. Ear defects can occur as a result of a variety of causes, including congenital conditions such as Microtia and acquired ones such as trauma, burns, and sports injuries [2, 3].

Several factors should be considered to enhance the better appearance of the reconstructed ear, with a focus on a sufficient suction system to ensure perfect skin coaptation to the framework without compromising overlying skin vascularity [4, 5].

There are various suction drainage tools, such as Jackson Pratt drains [6], test tubes [7], and syringes [8]. However, there is a knowledge gap about the optimal negative suction drainage system. There is no solid definition for ideal suction needed for auricular reconstruction but roughly, any suction device should apply a simple, safe, and consistent pressure to achieve a smooth auricular contour and at the same time to prevent collections i.e. seromas or hematomas [9, 10].

Another topic that needs to be investigated is the correlation between the appropriate suction pressure to be customized for any given patients’ age and skin type [6, 11]. This point should also be addressed thoroughly as oversuction can cause skin sloughing, while undersuction can lead to suboptimal results.

On the other side, Vacuum-assisted closure device (VAC) is a well-known tool in the plastic surgeon’s toolbox that is frequently used to treat complex wounds and skin defects [12, 13]. So, we postulated that it can be used as a suction tool i.e. continuous manner as a drainage system to obliterate dead space, maintain skin coaptation to the cartilage framework, absorb exudates and minimize complication rates.

## Patients and Methods

This is an interventional comparative study that was conducted in the Department of Plastic, Burn and Maxillofacial Surgery, Ain Shams University, Cairo, Egypt. Following the same Institutional Ethical Committee approval (FAMSU MS 689/2024). A total number of 20 patients were enlisted for this study with an age range from 6 to 12 years. We included all patients seeking auricular reconstruction for unilateral microtia. Patients with previous trials for reconstruction were excluded from the study. All surgical interventions in this study were done by the senior author.

All Studied patients were simply randomized into 2 groups; *Group I* (*n* = 10); The control group; for whom 50 ml Syringe was used as a suction tool and *Group II* (*n* = 10); The study group; Where the VAC device was used as a suction tool.

### Surgical Technique

**Fig. 1:**
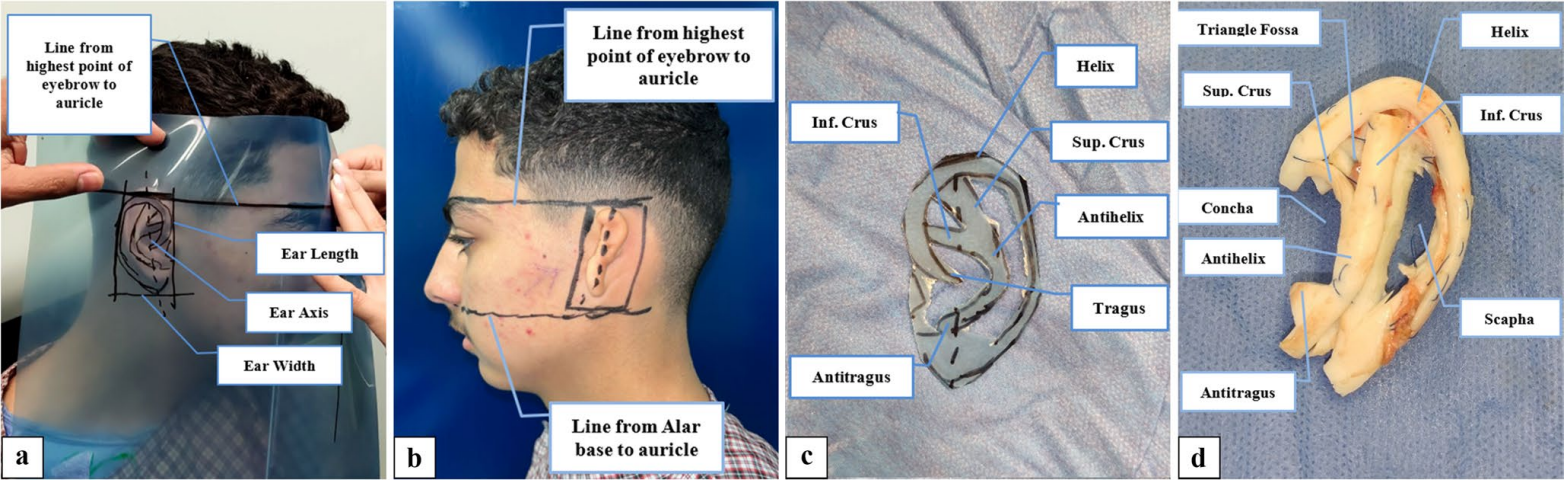
Making model for reconstructed ear; (**a**) represents a transparent film used to precisely mark the auricle’s position and inclination through line extended between highest point of eyebrows to highest point of auricle and lines represent ear length, width and axis are marked; (**b**) represents new auricle’s position through line from highest point of eyebrows to auricle and line from alar base to auricle; (**c**) represents model that used as a mirror the contralateral auricle; (**d**) represents fabrication of cartilage framework using Prolene 3/0 threads and mattress sutures with knots buried on the underside of the framework

Initially, a transparent film was used to mirror the contralateral auricle to accurately define the shape, position and inclination of the new auricle. Figure 1.

**Fig. 2:**
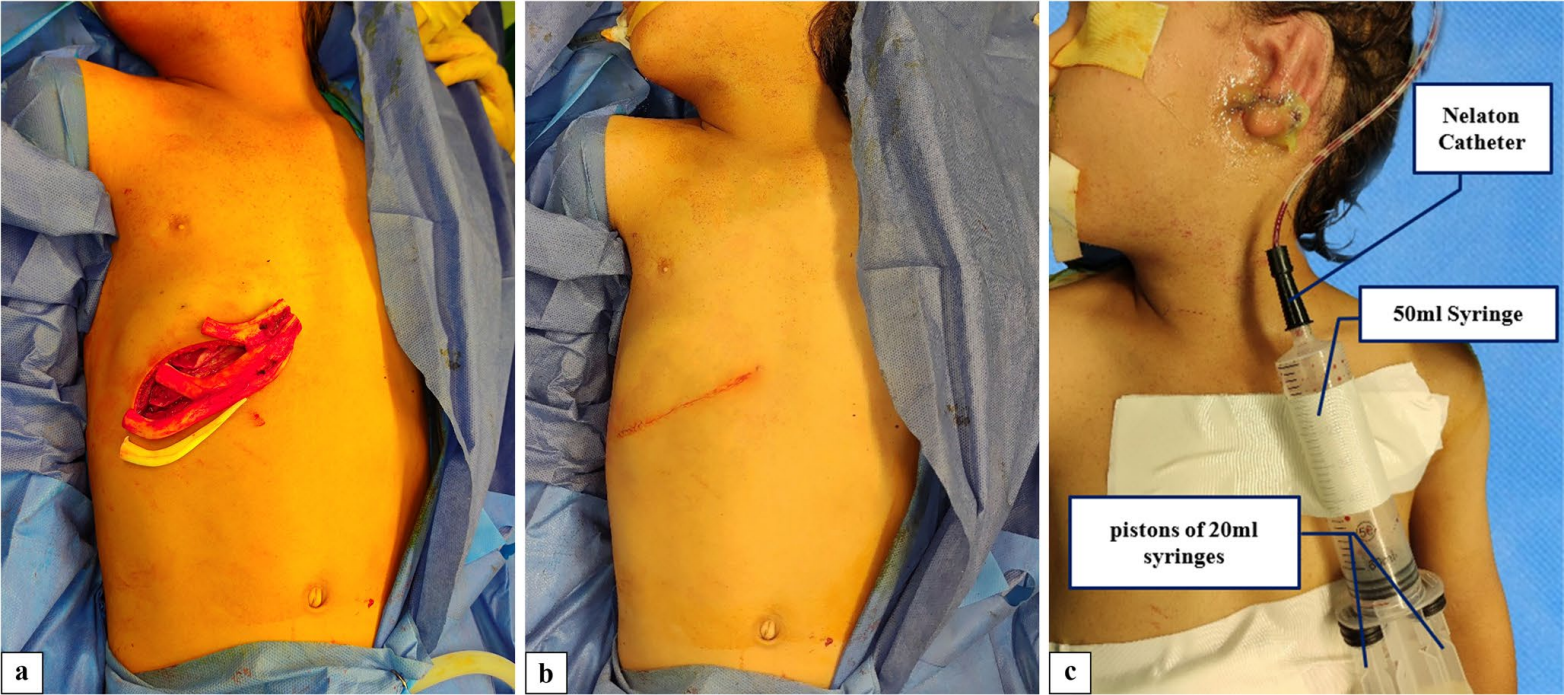
Donor site for cartilage stock; (**a**) represents harvesting the synchondrosis of the sixth, seventh, and eighth costal cartilages and the floating rib, via an oblique direct incision; (**b**) represents Skin donor closure in layers using Monocryl sutures; (**c**) represents Nelaton catheter 10 ^Fr^ placed in pocket and connected to 50 ml Syringe, then 2 pistons of 20 ml syringe were interposed to maintain the suction in Group I

Under general anesthesia. using an oblique direct incision, we harvest the right synchondrosis of the 6th, 7th, and 8th costal cartilages, as well as the floating rib. Then, using the prefabricated model as a guide, we fabricated the cartilage framework. Mattress sutures -Prolene 3 − 0 threads with straight needle- were used to assemble the framework, care should be taken to bury knots to the underside of the framework to prevent stitch sinus or skin sloughing. Figures 1 and 2.

Based on our experience, we try to minimize skin edema by delaying the creation of the retroauricular skin pocket after framework fabrication is complete. We also incorporate the lobular transposition in the first stage of auricular reconstruction.

**Fig. 3:**
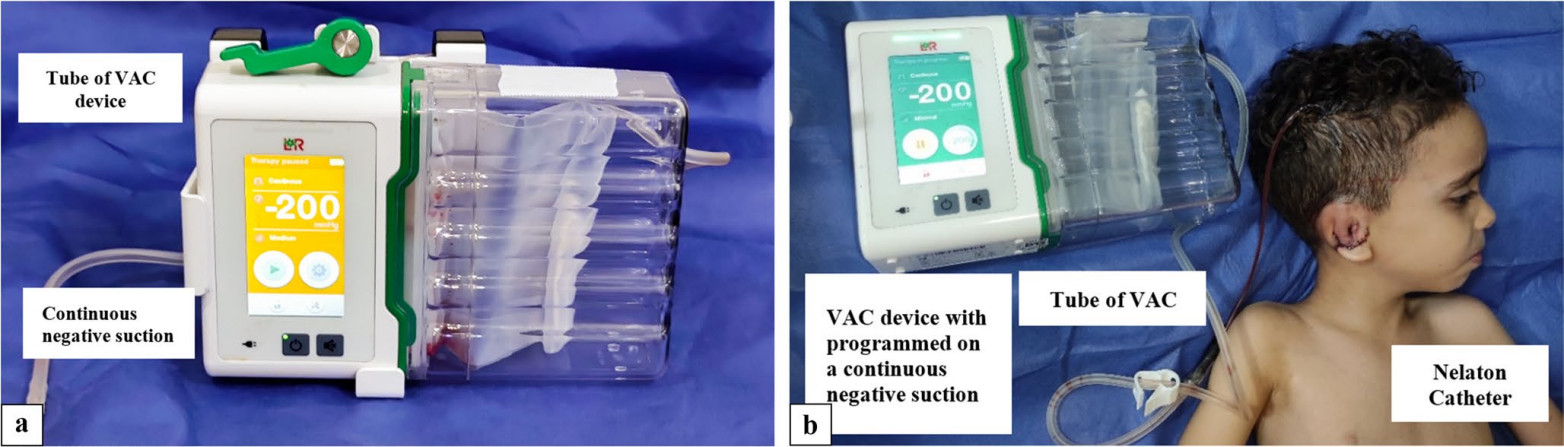
(**a**) represents a portable VAC device (Lohmann-Rauscher Suprasorb^®^ CNP P3 - German) used in Group II; (**b**) Nelaton catheter 10 ^Fr^ placed in pocket and connected to tube of VAC device programmed on a continuous negative suction to maintain the suction in Group II

Regarding suction method, Nelaton catheter 10 ^Fr^ was placed in pocket and In *Group I*; the catheter was connected to a 50 ml Syringe with interposition of 2 pistons of 20 ml syringe to maintain the suction Fig. 2. In *Group II*; a portable VAC device (Lohmann-Rauscher Suprasorb^®^ CNP P3 - German), was connected to the catheter on a continuous negative suction mode. The device pressure was set as the lowest pressure that precisely adhere the skin to the underlying framework without causing blanching or any signs of skin ischemia. Figure 3.

Post-operatively, Patients were admitted for two days after operation to monitor suction maintenance. In *Group I*, the syringe system was completely replaced after 10 ml collection. In *Group II*, the VAC device automatically adjusts the pressure to the desired level.

Upon discharge, the guardians were instructed to check the pressure maintenance and to consult the physician if they noticed any source of air leakage.

**Fig. 4:**
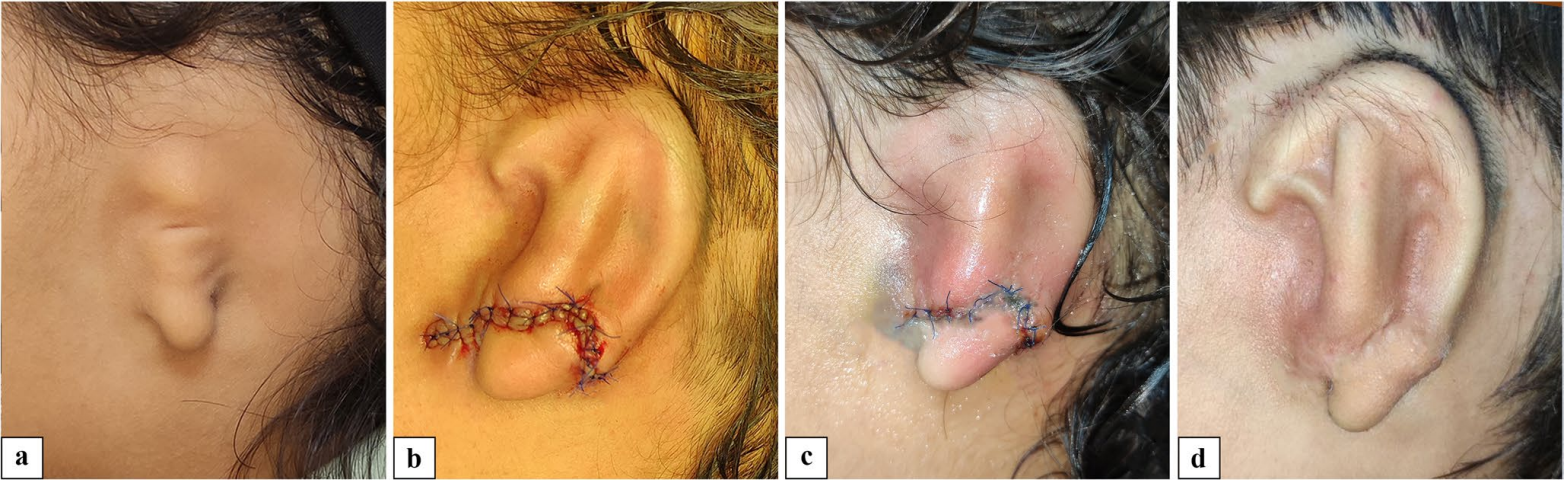
**a** represents a case of left microtia; **b** intra-operative photo of the case underwent autologous auricular reconstruction and used Syringe as a suction tool; **c** one week follow up; **d** six months follow up

**Fig. 5:**
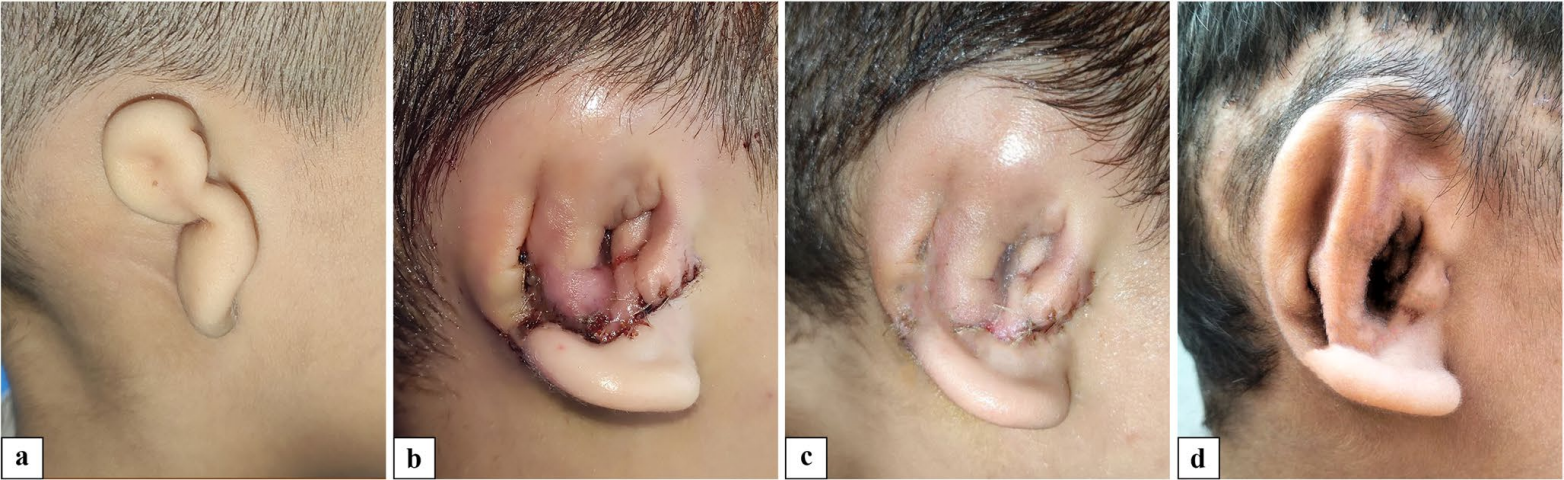
**a** represents a case of right microtia; **b** intra-operative photo of the case underwent autologous auricular reconstruction and used VAC as a suction tool; **c** one week follow up; **d** six months follow up

Follow-up visits are carried out one week after the procedure to detect the presence of postoperative complications, such as hematoma, seroma, exposed cartilage, and skin necrosis. Another visit was scheduled two weeks later for removal of whatever the suction device. Figures 4 and 5.

Six months after surgery, a questionnaire was performed to estimate patient satisfaction about the general appearance of the ear [14]. It was translated into Arabic and used a five-point Likert-type scale, with 1 denoting “very unsatisfied” and 5 denoting “very satisfied.”

Additionally, Pre and post photos of the patients were presented as a PowerPoint presentation in Prescence of 3 plastic surgery experts in ear reconstruction. A checklist was used to collect their opinions regarding the various aesthetic units of the reconstructed ear.

### Statistical Analysis

Results were presented as number and percentage for qualitative data or mean and standard deviation for quantitative data. Two sets of quantitative data were compared using Student’s t-test for dependent or independent means as appropriate. Chi square (or Fisher Exact) test was used to compare qualitative data between two groups. The results were considered statistically significant if P value was less than or equal to 0.05.

Statistical analysis was performed using computer software statistical package for the social science (SPSS, version 27; SPSS Inc., Chicago, Illinois, USA).

## Results

### Patient Characteristics

Mean age was 8.2 ± 2.42, with a range of 6–12 years. Regarding sex, the majority were males 75% while females represented only 25%.

### Total Patient’s Satisfaction Score

As shown in Table 1, that compares patient’s satisfaction between two methods. The mean total satisfaction score was significantly higher for the VAC group (22 ± 1.49) than syringe suction (18 ± 1.17) (*P* < 0.001). Specific item like Ear Contour consistently favored the VAC group, with significant P-values (*P* < 0.05).

**Table 1:** Comparison between two groups regarding patient’s satisfaction:

		VAC device (*N* = 10)		Syringe suction (*N* = 10)		t*	*P* value
		Mean	SD	Mean	SD		
Total patient’s satisfaction score		22	1.49	18	1.17	5.58	< 0.001
		N	%	N	%	X^2**^	P value
Ear Length	Neutral	0	0.00%	3	30.00%	4.63	0.12
	Satisfied	4	40.00%	5	50.00%		
	Highly satisfied	6	60.00%	2	20.00%		
Ear width	Unsatisfied	0	0.00%	2	20.00%	3.71	0.41
	Neutral	2	20.00%	4	40.00%		
	Satisfied	5	50.00%	3	30.00%		
	Highly satisfied	3	30.00%	1	10.00%		
Ear fitting with face	Neutral	0	0.00%	1	10.00%	1.55	0.65
	Satisfied	5	50.00%	6	60.00%		
	Highly satisfied	5	50.00%	3	30.00%		
Ear shape compared to normal one	Neutral	2	20.00%	3	30.00%	0.5	1
	Satisfied	4	40.00%	4	40.00%		
	Highly satisfied	4	40.00%	3	30.00%		
Ear Contour	Unsatisfied	0	0.00%	2	20.00%	14.41	< 0.001
	Neutral	0	0.00%	6	60.00%		
	Satisfied	4	40.00%	2	20.00%		
	Highly satisfied	6	60.00%	0	0.00%		

*Student’s t-test **Chi square test (FE: Fisher Exact)

Ease of Suction tool use consistently favored the VAC device, in addition there’s no air leakage detected in VAC group unlike syringe group, as shown in Table 2, consistently favored the VAC group, with significant P-values (*P* < 0.05). Regarding the device cost, Syringe group recorded higher satisfaction rate than VAC group, with consideration to individualized financial background and variability in patients’ perceptions of cost in relation to clinical benefits.

**Table 2:** Comparison between two groups regarding Suction tool during postoperative follow up:

Postoperative follow up		VAC device (*N* = 10)		Syringe suction (*N* = 10)		X^2*^	P value
		N	%	N	%		
Ease of Suction tool use	Very Difficult	0	0.0%	1	10.0%	19.40	< 0.001
	Difficult	0	0.0%	4	40.0%		
	Normal	0	0.0%	5	50.0%		
	Easy	3	30.0%	0	0.0%		
	Very Easy	7	70.0%	0	0.0%		
Suction tool Cost	Neutral	8	80.0%	0	0.0%	16.62	< 0.001
	Satisfied	2	20.0%	5	50.0%		
	Highly satisfied	0	0.0%	5	50.0%		
Suction problems (Air Leakage)	3 times	0	0.0%	2	20.0%	18.86	< 0.001
	2 times	0	0.0%	5	50.0%		
	one time	0	0.0%	3	30.0%		
	Minimal leakage	4	40.0%	0	0.0%		
	No Leakage	6	60.0%	0	0.0%		

*Chi square test (FE: Fisher Exact)

### Complications

The VAC device group had fewer complications. As 90% of VAC patients had no complications versus 70% in the syringe suction group (*P* = 0.26). The syringe group experienced more complications, including hematoma (20%), Skin necrosis & Exposed cartilage (10%), while the VAC group had only one case with Skin necrosis & Exposed cartilage noted (10%).

**Table 3:** Comparison between two groups regarding postoperative complications:

		VAC device (*N* = 10)		Syringe suction (*N* = 10)		X^2*^	P value
		N	%	N	%		
Postoperative Complications	No Complications	9	90.0%	7	70.0%	1.25	0.26
	Hematoma	0	0.0%	2	20.0%		
	Seroma	0	0.0%	0	0.0%		
	Infection	0	0.0%	0	0.0%		
	Hypertrophic scar	0	0.0%	0	0.0%		
	Skin necrosis & Exposed cartilage	1	10.0%	1	10.0%		

*Chi square test (FE: Fisher Exact)

### Experts’ Opinion

The next Table 4 compares experts’ opinion about aesthetic units of the ear between two methods at postoperative period, as Helix and lobule of reconstructed auricle get the highest level of satisfaction in both groups among other subunits of the ear. The mean total expert score was higher for VAC (31.4 ± 2) compared to syringe suction (25 ± 2.59) (*P* = 0.38). Sub-item analysis showed that experts rated the VAC group significantly better in aspects like ear contour and improving aesthetic results at postoperative period (*P* < 0.05).

**Table 4:** Comparison between two groups regarding experts’ opinion about aesthetic units of the ear at postoperative period:

Experts’ Opinion		VAC device (*N* = 10)		Syringe suction (*N* = 10)		t*	P value
		Mean	SD	Mean	SD		
		31.4	2	25	2.59	0.91	0.38
		N	%	N	%	X^2**^	P value
Helix	Neutral	0	0.0%	4	40.0%	10.33	0.01
	Satisfied	2	20.0%	5	50.0%		
	Highly satisfied	8	80.0%	1	10.0%		
Antihelix	Neutral	0	0.0%	3	30.0%	4.63	0.12
	Satisfied	4	40.0%	5	50.0%		
	Highly satisfied	6	60.0%	2	20.0%		
Concha	Unsatisfied	0	0.0%	2	20.0%	3.71	0.41
	Neutral	2	20.0%	4	40.0%		
	Satisfied	5	50.0%	3	30.0%		
	Highly satisfied	3	30.0%	1	10.0%		
Lobule	Neutral	0	0.0%	3	30.0%	6.41	0.04
	Satisfied	4	40.0%	6	60.0%		
	Highly satisfied	6	60.0%	1	10.0%		
Ear Contour	Neutral	0	0.0%	3	30.0%	5.75	0.04
	Satisfied	3	30.0%	5	50.0%		
	Highly satisfied	7	70.0%	2	20.0%		
Skin Color	Unsatisfied	0	0.0%	2	20.0%	3.71	0.41
	Neutral	2	20.0%	4	40.0%		
	Satisfied	5	50.0%	3	30.0%		
	Highly satisfied	3	30.0%	1	10.0%		
Improving aesthetic results at postoperative period	Unsatisfied	0	0.0%	3	30.0%	15.67	< 0.001
	Neutral	1	10.0%	7	70.0%		
	Satisfied	3	30.0%	0	0.0%		
	Highly satisfied	6	60.0%	0	0.0%		

*Student’s t-test **Chi square test (FE: Fisher Exact)

## Discussion

Securing a tight contact between the skin flap and the implanted cartilage framework is crucial for ear reconstruction for microtia in order to achieve an accentuated contour; this also reduces dead space and avoids the formation of hematomas [7, 15].

In contrast to Brent [16], who used an X-ray film for preoperative planning based on the opposite normal ear, and cut with a scalpel to create a model that could injure the surgeon, our study uses a thinner transparent film with a chart to help with dimension accuracy and for easy cutting without endangering the surgeon.

Negative suction drainage is frequently employed to prevent seromas or hematomas in auricular reconstructions; however, there are few articles that discuss negative suction. We believe that there is worthy information regarding negative suction that is essential for a healthy skin-framework occlusion [11, 17].

Following first stage auricular reconstruction, various suction techniques have historically been employed. For example, Brent [16] used a commercially available but somewhat expensive vacuum test tube, Park DH et al. [7] used a negative suction system that included a disposable syringe, and a wooden tongue depressor, and Durrani AJ et al. [6] used two Jackson Pratt-type silicone drains. Additionally, Xu Z et al. [8] used two tiny polyethylene drains that were connected to a 20 mL disposable syringe that had a needle cover and a piston.

In contrast to other traditional methods that provide a standard pressure which is inappropriate for different cases, our study used a sample suction method that involves a VAC device connected to a Nelaton catheter sited within the skin pocket through a single incision rather than two incisions as in Xu Z and Durrani AJ. Additionally, the VAC device allows us to modify the negative pressure applied for each individual age and skin type to suit the current state of the skin flap.

According to Park DH et al. [7], the wooden tongue depressor length was adjusted to change the pressure, which is inaccurate for generating a safe and appropriate pressure, results in suction loss, and requires repeated attempts to achieve the right suction.

According to Xu Z et al. [8], 3 cases had an abnormally high volume of serum exudate during the first 2 postoperative days. In these cases, 20 mL syringes were replaced by 50 mL syringes, which were changed every 30 min. According to Brent, vacutainer tubes need to be changed every 4 h or less because the drain system clots. In contrast, our method eliminates the need to replace any part of the suction system because a large canister that is integrated with the VAC device allows for the receipt of any volume of exudate up to 1000 ml.

In contrast to syringes, which may lose suction without the caregiver’s awareness, our method detects air leakage by properly monitoring any source of leakage and automatically attempting to restore the functional suction again. Additionally, it has an automatic locking feature that keeps patients from making mistakes when using the device, which can easily happen with syringes, and at the bedside it provides an audio and visual alarm in case of air leakage for quick interaction and adjustment from the nursing team.

Unlike syringes with objective measurement, VAC devices provide an accurate measurement of volume exudate, which aids in determining when to remove the suction system and, consequently, shortens patient hospital stays.

We identified a drawback in the VAC device’s comparatively large size when compared to more conventional techniques like syringes, but it was still lightweight, portable, and didn’t restrict the patient’s range of motion. Additionally, caregiver satisfaction ratings for the device’s usability were higher than those for syringes.

Another limitation of the study is regarding device-related costs, as caregivers in the syringe group reported higher satisfaction rates, likely reflecting the lower upfront expense of syringes. Although caregivers noted that VAC devices were considerably more expensive, many expressed satisfaction when the cost was viewed in relation to the clinical benefits and improved outcomes associated with VAC therapy. However, it is important to acknowledge that perceptions of cost-effectiveness may vary among families depending on their socioeconomic background, and therefore these findings should not be generalized without considering this variability.

Further research is needed to compare different pressure measurements needed for each age and skin type without depending on a suction method with inaccuracy of pressure.

## Conclusion

The VAC device is a safe and effective adjunct for optimizing surgical outcomes in the first stage of autologous auricular reconstruction. Its application promotes consistent contouring, reduces complications, and enhances patient satisfaction. Further studies are warranted to validate long-term benefits.
